# Emotional Sentence Annotation Helps Predict Fiction Genre

**DOI:** 10.1371/journal.pone.0141922

**Published:** 2015-11-02

**Authors:** Spyridon Samothrakis, Maria Fasli

**Affiliations:** Institute for Analytics and Data Science, University of Essex, Wivenhoe Park, Colchester CO4 3SQ, United Kingdom; Jiangnan University, CHINA

## Abstract

Fiction, a prime form of entertainment, has evolved into multiple *genres* which one can broadly attribute to different forms of stories. In this paper, we examine the hypothesis that works of fiction can be characterised by the emotions they portray. To investigate this hypothesis, we use the work of fictions in the Project Gutenberg and we attribute basic emotional content to each individual sentence using Ekman’s model. A time-smoothed version of the emotional content for each basic emotion is used to train extremely randomized trees. We show through 10-fold Cross-Validation that the emotional content of each work of fiction can help identify each genre with significantly higher probability than random. We also show that the most important differentiator between genre novels is fear.

## Introduction

Fiction forms the basis of most modern forms of entertainment; works of fiction are often “translated” into games and movies, providing a formidable substrate of stories on which one can share unique experiences. One of the most important elements of a story is the rhetoric [1] it uses to elicit different levels of emotion to its readers. Emotion seems to be fundamental to human behaviour and studies have shown that damage in emotionally-charged neural pathways causes severe issues in decision making [2]. Emotional responses to fiction go beyond the simple hedonistic outlook prevalent in modern life; this allows the reader to emmerse herself in a fictional environment and potentially (re-)live and (re-)experience a life that is not her own.

Textual analysis of emotion has mostly focused on sentiment analysis, which attributes valence (i.e., positive or negative feelings) to text snippets [3]. Alongside this arguably one-dimensional view of human emotions, models have been developed that try to capture all six basic emotions (i.e., anger, disgust, fear, joy, sadness, surprise) [4] in text. In the process, a popular database of emotionally-charged (e.g. “hate”) synonyms (“synsets”) has been identified and labelled Wordnet-Affect [5]. Synsets are synonym rings that form Wordnet [6], a large online thesaurus. Previous work in analysing the emotions prevalent in text has focused on analysing news headlines and blog posts [7, 8] and books in time [9]. Blog texts in particular have been used [10] to help identify the mood of authors when writing each post. Though classification results over the baseline (over 0.5 precision) are not spectacular, such methods are clearly viable. In the classic task of movie review sentiment analysis [11] for example) accuracy rates of over 0.83 are achievable.

As a written work of fiction (essentially a long piece of text) tries to convey emotions, it is natural to assume that it can be analysed using text analysis techniques and also postulate that different types of fiction portray different emotions, both in terms of time (i.e. which portion of a work of fiction portrays what) and in terms of type (what emotions are portrayed in what works of fiction). This forms our main hypothesis and the basis of this study. In this study, we identify the emotional content of sentences, move from sentences to novels and try to predict the genre of a work of fiction based on this analysis of its emotional content. To achieve this we train extremely randomized trees and compare with baseline classifiers. Results show a huge boost in predictive power compared to both stratified and most frequent class baseline classifiers.

Though (to the best of our knowledge) this is the first study trying to link emotions portrayed in fiction with the fiction genre, there are some peripheral studies worth mentioning. Bentley et. al. [12] followed an approach similar to the one used here, but on a huge historical corpus provided by google online and showed a strong correlation between economic performance and the use of negatively charged emotions. Hughes [13] et.al. use texts available in project Gutenberg to show that within certain time periods literary styles happen to coincide. In the realm of sentiment analysis, one can find similar work of a descriptive nature being done by Jockers [14], but no direct link to genre is given, albeit it should be straightforward to extend it. Within the context of Songs, blogs and speeches, Dodds and Danforth [15] are able to correlate author age and time at the time of writing with the happiness level of each piece of work. Finally, the closest piece of work we could identify to ours [16] is a quantitative and visualisation study on the role of emotions in fiction, but with no correlation with genre.

## Materials and Methods

Our basic hypothesis is that different genres of fiction elicit different emotions, and thus one can predict the genre of a work of fiction through its emotional content. In this section we describe how and what was collected alongside how we did the processing.

### Materials

We used Project Gutenberg [17] as a means to collect freely available works of fiction, categorised by individual experts. The genres, numbers and ids can be seen in Table 1. We performed a simple keyword search for each category and collected 3403 works in total. The breakdown for each genre can be seen in Table 1.

**Table 1:** List of genres, numbers of instances found and unique ids for each class (i.e, genre).

mystery	648	0
humor	662	1
fantasy	346	2
horror	108	3
science fiction	1252	4
western	387	5

The materials were collected and cleaned up by removing copyright and Project Gutenberg notices. We went through the whole of project Gutenberg catalogue and searched for the following keywords under the **subject** metadata: “science fiction”, “horror”, “western”, “fantasy“, “crime fiction”, “mystery”, “humor”, “romance”. Romance novels where discarded as very few were found (< 20). We also removed all copies of the humoristic journal London Charivari.

### Emotional Content of fiction

We measure the emotional content of a sentence using a simple approach termed WN-affect presence [8], which simply adds up the number of Synsets (i.e. rings of synonyms) in each sentence that are attributed to an emotion as provided by Wordnet-Affect. We use the Natural Language Toolkit in Python [18] to extract the relevant information for each sentence. Thus each sentence will get six real numbers, one for each basic emotion. Note that the presence of words is the only thing we take into consideration when calculating the emotional content of a sentence, which is arguably the simplest method possible. It obviously does not take into account many of the intricacies of text, but it seems to work relatively well in practice, performing reasonably well as has been demonstrated in previous studies [8]. Wordnet-Affect had precision scores of 0.33, 0, 100.0, 50, 33, 13.4 for anger, disgust, fear, joy, sadness, surprise. Recall rates were 12.8, −1.59, 24.86, 10.32, 8.56, 3.06 respectively. In the last four emotions, these precision scores ranked top among four different methods in the task of emotional news headline classification. The algorithm had low recall and performed badly on disgust (though this could have been due to a data bias). If we are to take into account that no overall “best” method exists, choosing a simple method that performs reasonably well as a “feature extractor” seems reasonable.

We proceed by repeating the process for each sentence of each work of fiction. Thus initially, each work is transformed into six separate signals, one for each basic emotion. The signals change through time as the emotional content of the sentences of fiction changes. Each signal is smoothed using a Hanning smoother [19] with window length being the total length of the signal divided by three, *M* = ∣*tss*∣/3, where *tss* is the signal vector for the whole text. Three was chosen heuristically, however the important fact to note is that the smoothing is proportional to the total signal window, as signals have different sizes. The window smoother thus is as in Eq 1

w(n)=0.5-0.5cos((2πn)/(M-1)) (1)

**Fig 1:**
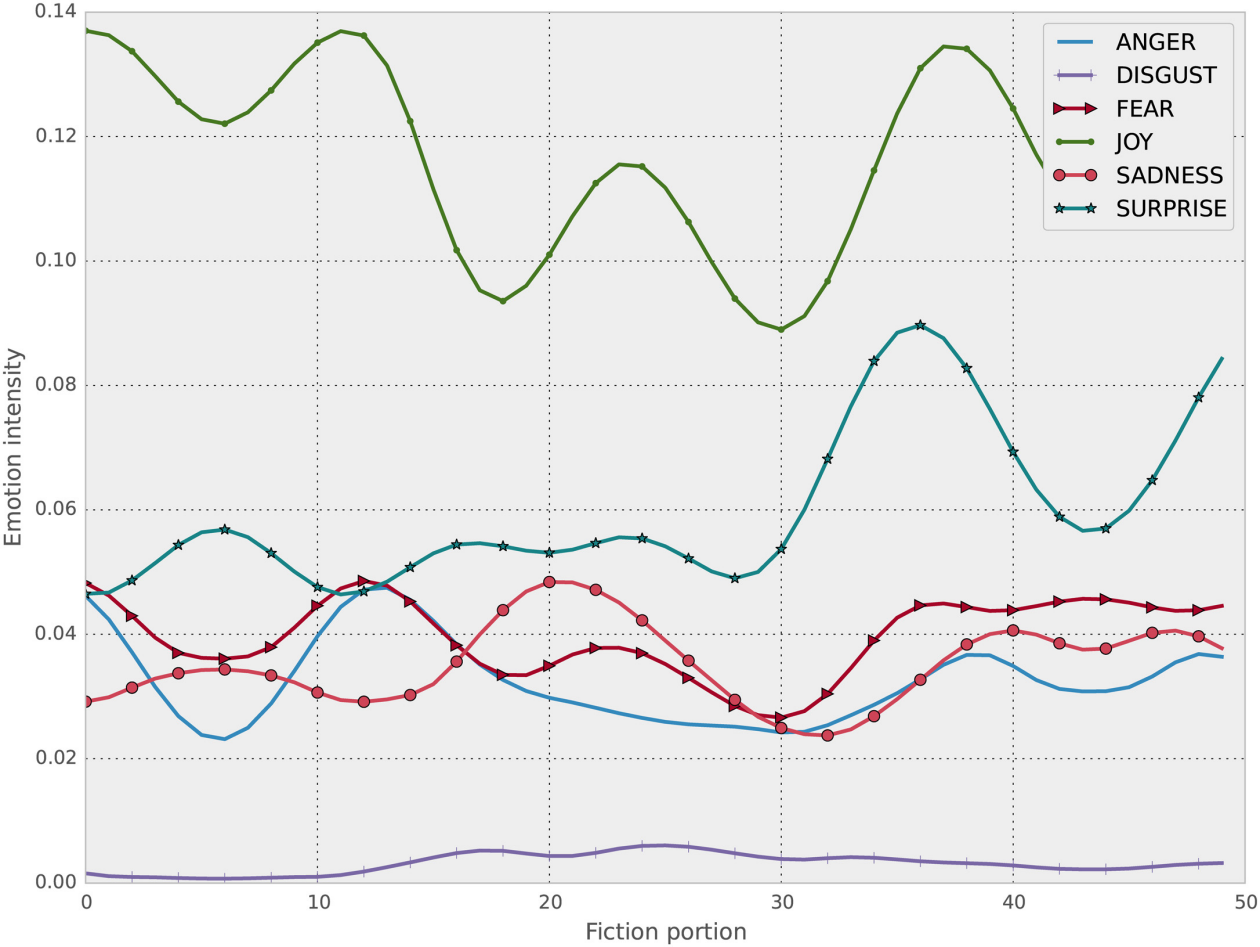
Emotional content for Murder at Bridge, by Anne Austin, of class Mystery.

**Fig 2:**
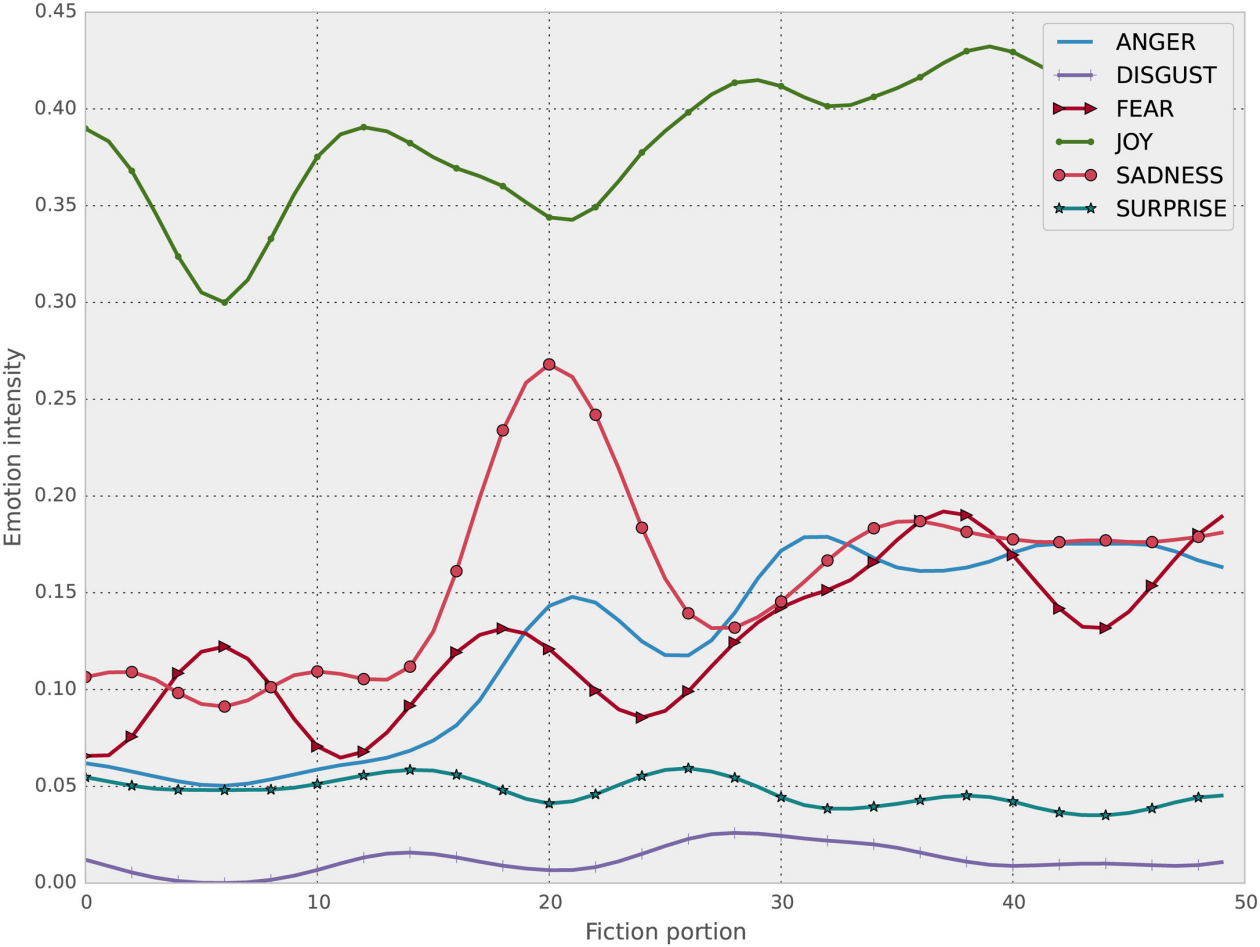
Emotional content for Frankenstein, by Mary W. Shelley, of class Horror.

We then take the average of the signal for each *n* sentences, where ∣*tss*∣/*c*, where *c* = 50 is a constant. This in effect creates a smoothed version of the signals with 50 timesteps, no matter how big the original signal length (i.e, the size of the work of fiction) was. This however meant that some works that had less than 50 sentences in total had to be removed, which brought down the total sample size to 3377. Sample signals of this type for all emotions can be seen for two novels in Figs 1 and 2. Notice the continuous fluctuation of signal strength. The smoothing/averaging process described has the explicit goal of turning a very noisy signal to a version that can be fed into a classifier and that minor differences are removed. The noise comes from multiple sources (e.g., errors in the emotional content analysis) but the size of the overall text allows for the total feeling to be captured.

In total, we analysed 3377 individual works of fiction and we extracted 300 features for each work of fiction, 50 for each basic emotion.

## Results

**Fig 3:**
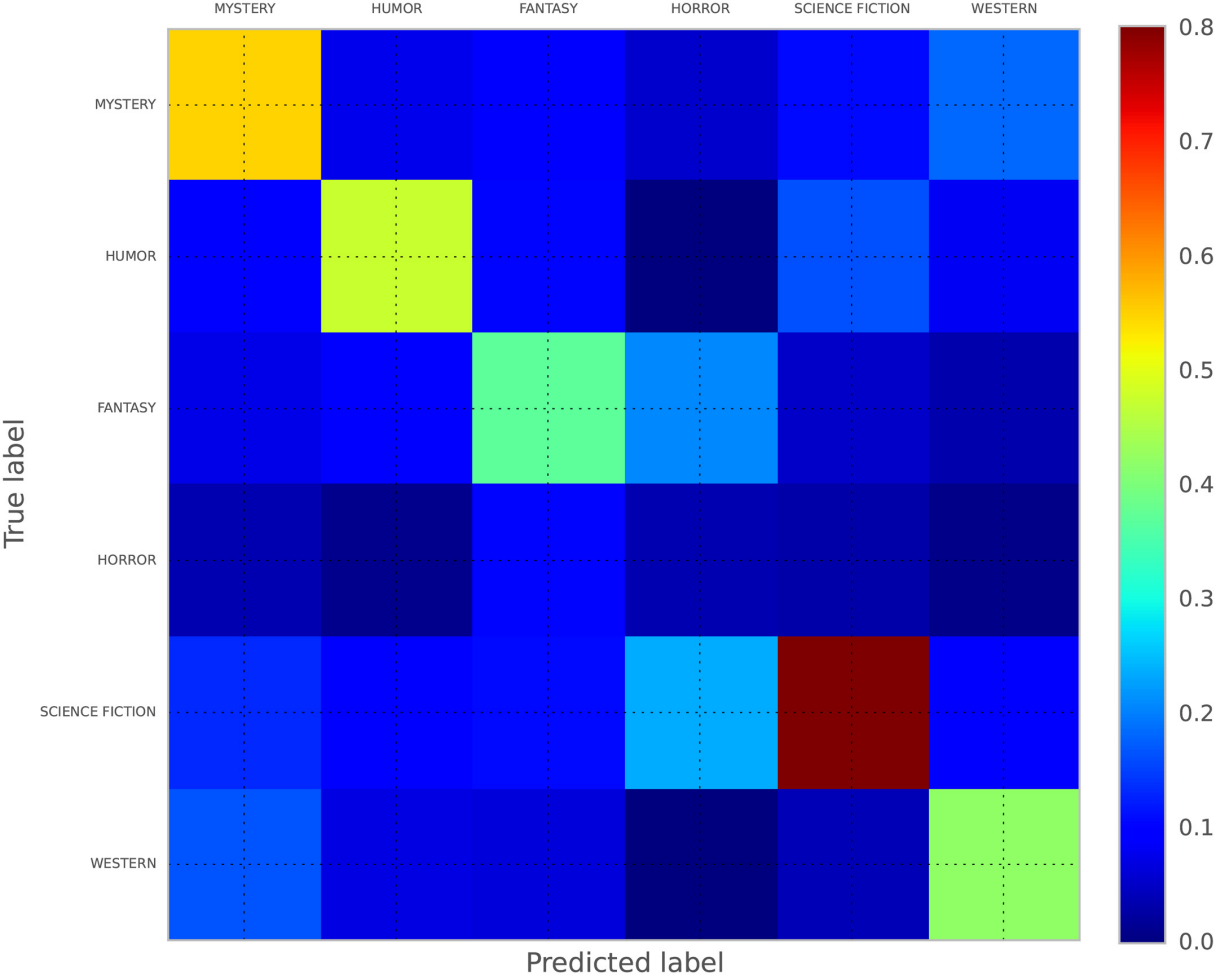
Scaled confusion matrix.

We perform 10-fold cross-validation using all samples, as this was shown empirically to have an optimal bias-variance tradeoff [20]. We train extremely random forests [21] using scikit-learn’s Python implementation [22] of the algorithm. We use 1500 trees. A scaled average version (between [0, 1]) of all the confusion matrices of the results can be seen in Fig 3 and the results are available in Table 2. The stratified classifier simply chooses labels proportionally to the instances represented in the data. It creates a multinomial distribution based on the training labels and samples from it in order to predict the correct label. The most frequent classifier predicts the class with the highest number of instances. One can clearly see that using emotions can help almost double accuracy performance. In our case, accuracy is measured as: $accuracy\left(genre,predicted\_genre\right)=1/3377\sum_{0}^{3376}1\{genre=predicted\_genre\}$, where 1 is the indicator function.

**Table 2:** Baseline classifiers and extremely random forests accuracy. Baseline classifiers performance is on the whole training set.

Most Frequent	0.369558779982
Stratified	0.223275096239
Extremely Random Forests	0.579646017699

**Fig 4:**
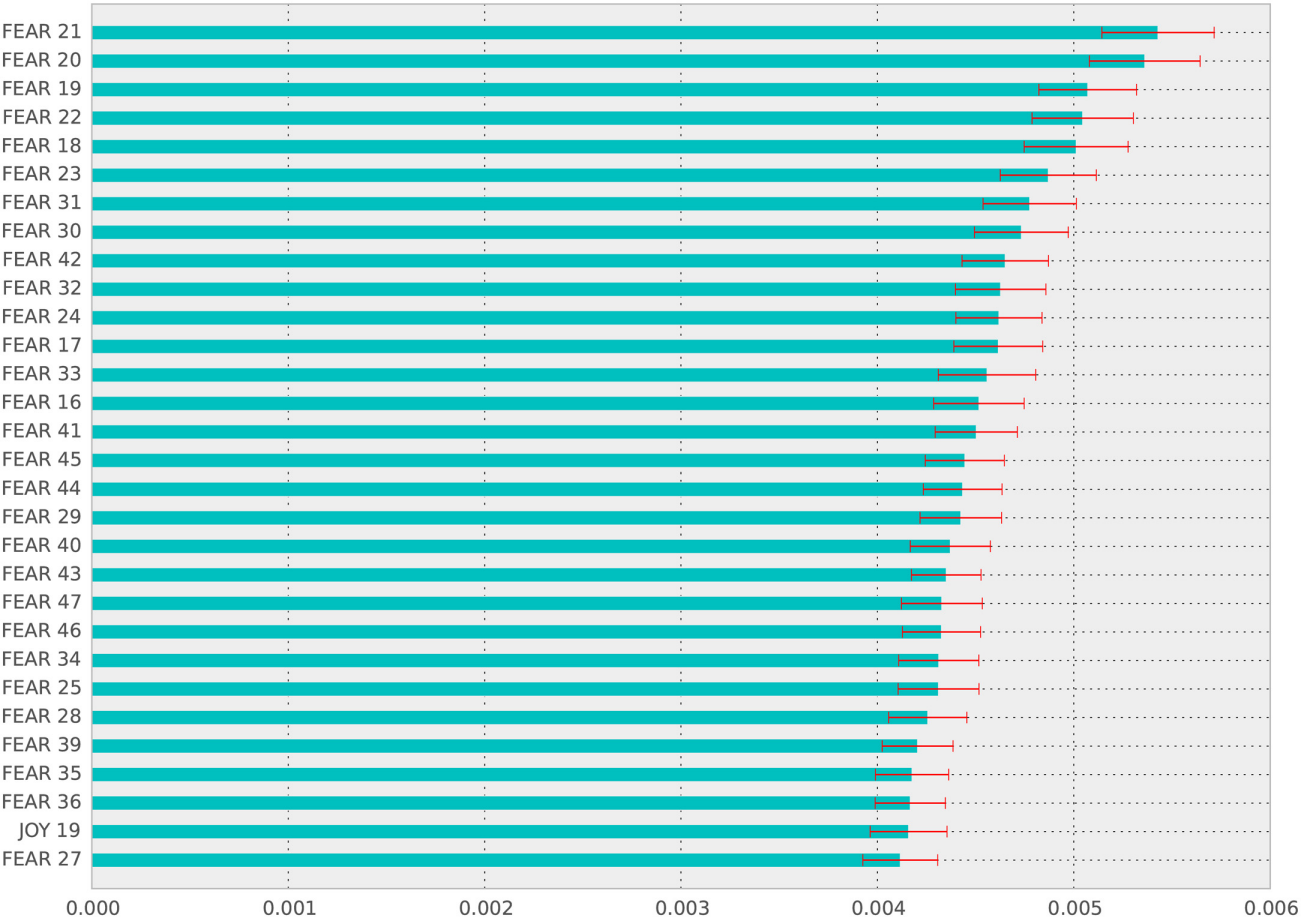
Feature importances, with *a* = 0.05 confidence intervals bars.

As evident in the confusion matrix, the classifier mostly missclassifies horror fiction as either fantasy or science fiction. The biggest proportion of correctly classified fiction is science fiction, followed up by humor, which also make up the bulk of our samples. We calculate the importance of each feature and present it in Fig 4. Each tree used by the classifier attributes its own split importance to features and the average of this split importance is plotted alongside the 95% confidence interval. We can see that the most discriminating features is fear.

**Fig 5:**
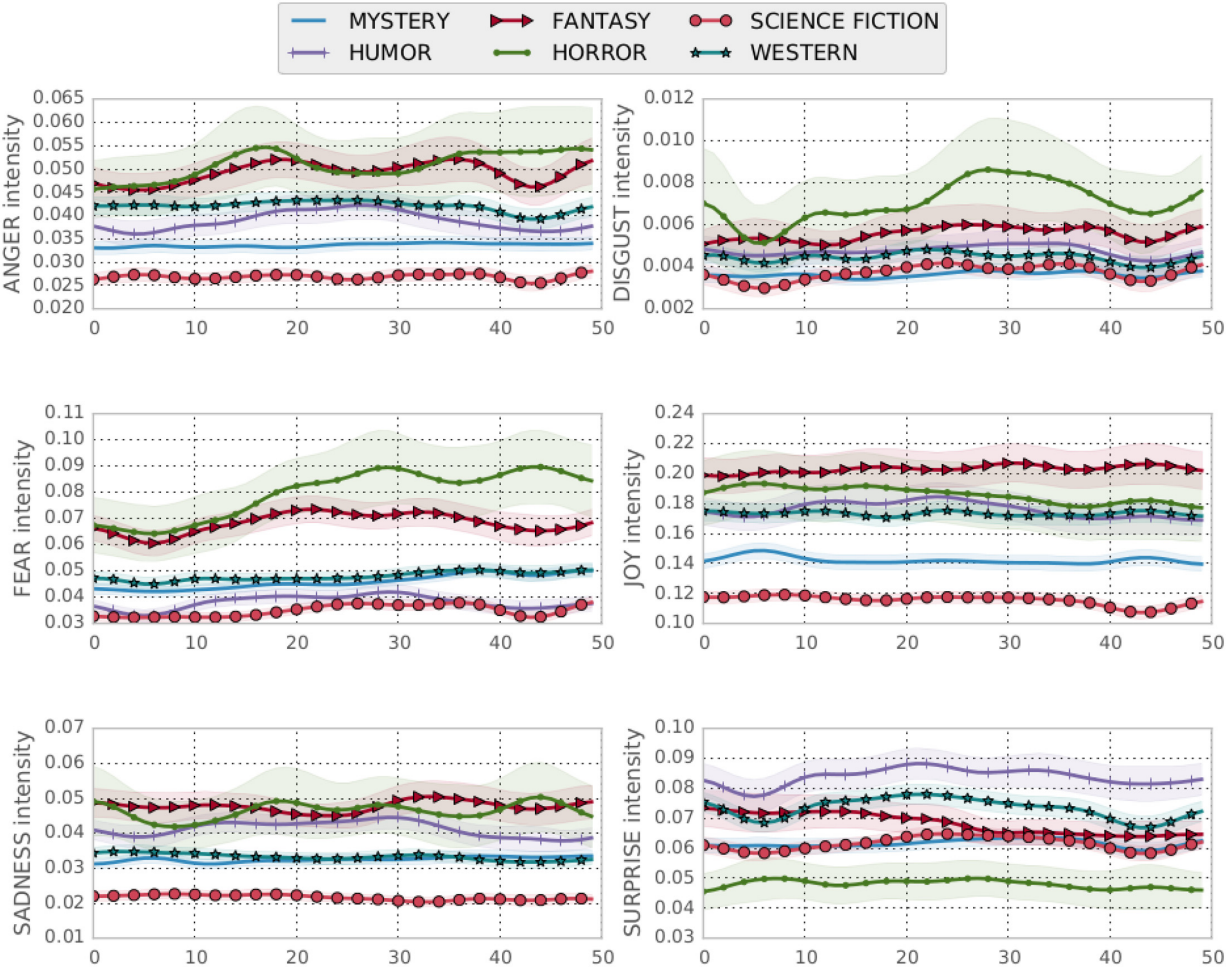
Average strength of each emotion among all texts. Statistical significance for *a* = 0.05.

It is interesting to observe that averages (as portrayed in Fig 5) cannot be correlated with genres, although some averages make intuitive sense (e.g., high fear and disgust values in horror fiction). Overall it seems that horror novels have higher “emotional content”, with the fear element spiking in the last part of the novels. On the other hand, Science Fiction novels seem comparatively low on emotions. Humor seems to be based heavily on suprise keywords. Another interesting result that clearly stands out is how strong the Joy element is in all texts, relatively to other emotions. This might be due to the higher number of keywords related to joy compared to the rest of the emotions. Overall however, the “time element” of emotions needs further clarification, and possibly a study on more novels, in order to be able to draw more representative conclusions.

The results we present are simple and portray a clear relationship between emotions and genre. This relationship however between the emotional content and the genre is somewhat lost within the complex non-linearities of the trees used. To amend this we summarised the whole text with just a single variable, and though there is a drop in performance using trees (accuracy is ≈ 0.54), the strength is still significant. We can now fit a linear function approximator that can be analysed intuitively. Accepting a further drop in performance (this time accuracy is ≈ 0.42), using logistic regression in conjunction with “l1” regularisation, we can obtain coefficients for each emotion (see Table 3). From Table 3 we can draw some interesting conclusions; humor fiction is negatively correlated with fear, but has a positive correlation with surprise, while horror and fantasy hole a strong positive correlation. On the other hand, science fiction has a negative correlation with joy. This analysis further demonstrates fear as a discriminatory variable for works of fiction.

**Table 3:** Emotion importance matrix.

	anger	disgust	fear	joy	sadness	surprise
mystery	-0.066	-0.093	0.152	-0.145	0.114	-0.052
humor	0.069	0.014	-0.56	0.282	0.248	0.432
fantasy	-0.003	0	0.261	0.051	0.158	-0.082
horror	0.017	0.064	0.27	-0.094	0.055	-0.182
science fiction	-0.114	0.082	0	-0.222	-0.472	0.04
western	0.179	-0.015	-0.082	0.145	-0.15	-0.007

## Discussion

This research was motivated by the hypothesis that works of fiction can be characterised by their emotional content and emotions can therefore be used to predict genre. We have deployed and presented the results of a simple method for correlating the emotions portrayed in works of fiction with the genre of the work. Our experiments using the Project Gutenberg works demonstrate that the use of emotions can indeed be used to predict genre with significant accuracy. As far as we are aware, this analysis of fiction texts based on emotions has not been undertaken before. We have also shown that the most important emotion, when it comes to classifying texts, is fear.

There is a number of important future directions one can take as part of this research. The most important one is analyse more fiction sources and vastly increase the size of the data. This would allow for a much more comprehensive study to take place and would extend the results of this limited-in-size study. Coupled with vast amounts of works of fiction, one can possibly try to analyse how genres correlate with the time they were published and the relationship of emotions to sales and user ratings, linking this work to previous studies [13].

Another important follow-up of this research is treating the hyperparameters used in this study (i.e, the smoothing/averaging process and their configuration) as variables and conduct an extensive study as to the impact of these variables in the quality of the learning.

Another possible avenue for exploration would be to increase the number of emotion keywords used (and thus the link between text and each book), possibly by introducing the dataset used by Saif [16]. Finally, a completely different direction would be to use an n-gram model (e.g., in the form of recurrent neural networks) to help identify genres.

All the above extensions however require access to vast quantities of digitised works of fiction and proper annotation of their genre, something that might not be publicly available for the foreseeable future. What we have done in this study is establish a new problem and create a baseline that is open up to further study in an extremely interesting topic.
